# The impact of the radical urbanization index on depression risk among middle-aged and elderly Chinese: Evidence from a large-scale cross-sectional study

**DOI:** 10.1371/journal.pone.0338329

**Published:** 2026-04-03

**Authors:** Yuting Li, Diege Long, Zhen Ye

**Affiliations:** 1 Longquan Hospital of Chengdu University of Traditional Chinese Medicine, Sichuan, Chengdu, China; 2 People’s Liberation Army (PLA) Western Theater Command General Hospital, Sichuan, Chengdu, China; 3 Shangrao Guangxin District People’s Hospital, Jiangxi, Shangrao, China; 4 Jiangxi University of Chinese Medicine, Nanchang, China; South China Normal University, CHINA

## Abstract

**Background:**

Rapid urbanization in China, while boosting socioeconomic development, has triggered “radical urbanization” characterized by land expansion outpacing population growth and economic activity. This spatial mismatch manifests as high building vacancy rates and underutilized infrastructure. Although urbanization is linked to mental health outcomes, evidence on radical urbanization’s impact on depression—especially among middle-aged and elderly populations—remains scarce.

**Methods:**

This cross-sectional study utilized 2011 baseline data from the China Health and Retirement Longitudinal Study (CHARLS), including 11,126 participants aged ≥45 years. Depressive symptoms were assessed using the CES-D scale (score ≥10 = clinically significant). The radical urbanization index was calculated as the log ratio of city-level built-up area (from China City Statistical Yearbook) to aggregated nighttime light DN values (using thresholds: eastern China ≥34, western China ≥25). Multivariable logistic regression (three adjusted models) and restricted cubic splines evaluated associations. Subgroup analyses and machine learning models (AUC comparison) further validated findings.

**Results:**

(1) Radical urbanization index was significantly higher in the depressive symptoms (DS) group vs. non-depressive symptoms (NDS) group (−4.74 vs. −4.96, *p* < 0.001). (2) Each 1-unit increase in the index elevated depression risk by 26% (OR=1.26, 95% CI:1.14–1.38; fully adjusted Model 3). (3) A dose-response relationship emerged: Q4 (highest index) had 1.68 × higher depression risk than Q1 (lowest) (*p*-trend<0.001). (4) Stronger associations occurred in central/western China (e.g., Hunan, Sichuan), low-education groups (OR=1.31), and rural residents (OR=1.30). (5) Adding the index to prediction models improved test-set AUC from 0.649 to 0.663.

**Conclusion:**

Radical urbanization—reflecting inefficient land-use and economic-population mismatch—is independently associated with depression among Chinese middle-aged and elderly adults. Findings underscore the need to reorient urbanization policies from “land expansion” to “human-centered” development to mitigate mental health burdens.

## 1. Introduction

Depression has emerged as a global public health issue, severely affecting patients’ quality of life. According to the 2019 Global Burden of Disease (GBD) data, the number of individuals with mental disorders worldwide surged from 650 million in 1990–970 million in 2019, marking a 48.1% increase over three decades [1]. Depression is a leading cause of mental health-related disease burden globally and ranks among the primary contributors to disability among adults, linked to conditions such as frailty, falls, dementia, and suicide [2–4]. With the escalating challenges of an aging society, the prevalence of depression among middle-aged and elderly populations is on the rise.

In China, the rapid urbanization since the reform and opening-up period—reaching an urbanization rate of 60.6% in 2019 — has introduced unique mental health challenges [5]. While urbanization has improved personal income, education, healthcare, and infrastructure [6,7]，the rapid and unplanned urbanization process may also heighten the risk of depression due to factors such as poor healthcare quality, economic pressure, and environmental hazards [6,8]. A key manifestation of this inefficiency is the phenomenon of radical urbanization, which we define as a mismatch among land expansion, population growth, and economic vitality. It is characterized by the expansion of built-up areas outpacing urban population growth, leading to high vacancy rates, underutilized infrastructure, and inefficient resource allocation. In recent years, several studies have explored the association between urbanization and depression. For adolescents, urbanization has a positive impact on their mental health, and female adolescents are more susceptible to depression than male adolescents [9]. Excessively high or low levels of urbanization may lead to physiological or psychological health issues among middle-aged and elderly individuals [10]. Moreover, environmental pollution, changes in greenery, nighttime lighting, and sleep disorders resulting from urbanization have all contributed to the incidence of depression [11–13]. Although previous research has documented the relationship between urbanization and depression, most of these studies have been region-specific and primarily focused on observing differences between urban and rural areas [14,15]. To date, researchers have employed various indicators to assess urbanization levels, such as the urban-rural dichotomy [6], nighttime light index [16], population density [17] and gross domestic product (GDP) [18]. However, these measures have limitations: the urban-rural dichotomy overlooks transitional zones and internal heterogeneity; the nighttime light index reflects economic activity but fails to capture land-population mismatch; and population density quantifies aggregation but struggles to reveal resource allocation efficiency.This study utilizes a radical urbanization index that integrates remote sensing satellite nighttime light data (a proxy for economic vitality) and built-up area data (a proxy for physical expansion) to construct a comprehensive measure of the “land-population-economy” triple mismatch. This index captures the phenomenon of “land urbanization without population aggregation,” a distinctive issue in China. If this form of low-quality urbanization persists, it may lead to sustained spatial disorder and broader social health challenges. If this low-quality urbanization model, dominated by land sprawl and urban scale expansion, is not fundamentally transformed, issues such as disordered urban spatial expansion and inefficient resource allocation will persist, ultimately hindering economic development and posing social health challenges. Nevertheless, evidence linking radical urbanization to depression remains scarce.

Understanding the link between rapid urbanization and depression among middle-aged and elderly populations is crucial for developing targeted strategies to mitigate depression risks amid ongoing rapid urbanization and demographic shifts. To delve deeper into the association between radical urbanization and depression, this study employs data from the China Health and Retirement Longitudinal Study (CHARLS), adopting a prospective cohort study design to analyze the relationship between the radical urbanization index in different regions and the outcomes of depressive symptoms among the population. CHARLS is a nationally representative longitudinal survey with robust follow-up data and quality control, providing a vital foundation for this research.This study contributes to a deeper understanding of the association between radical urbanization and depression, offering scientific evidence for formulating prevention and intervention strategies tailored to the current situation of depression among middle-aged and elderly individuals in China.

## 2. Method

### 2.1. Data source and study population

CHARLS is a prospective study conducted among individuals aged over 45 in 28 provinces across China [19]. The nationally representative CHARLS data were collected via face-to-face interviews using standardized questionnaires, covering demographics, lifestyle, and health status.Ethical approval for CHARLS was obtained from the Biomedical Ethics Review Committee of Peking University (IRB00001052–11015), and all participants provided written informed consent.

**Fig 1:**
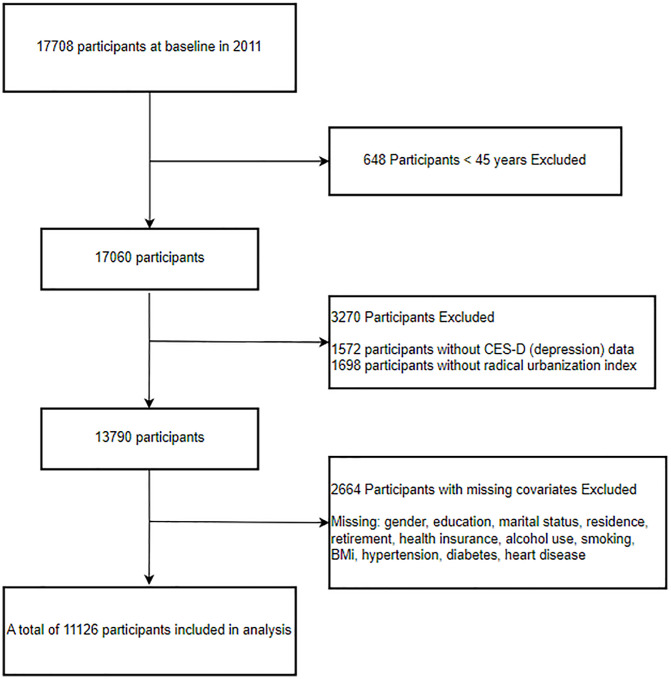
Flowchart for study population selection in the CHARLS database.

This study is a cross-sectional one that utilized baseline data from 2011, incorporating a total of 17,708 participants. Initially, due to sample design issues, some data might have included young individuals under 45 years old, so we excluded participants below this age threshold (648 participants). Secondly, we excluded participants with missing data on exposure factors and outcome variables (1,572 participants had missing Center for Epidemiologic Studies Depression Scale (CES-D) scores, and 1,698 participants had missing radical urbanization index data for their respective cities). Thirdly, we excluded 2,664 participants with missing covariate data (including gender, education level, marital status, living situation, retirement status, medical insurance coverage, alcohol consumption history, smoking history, body mass index (BMI), as well as the presence of hypertension, diabetes, and heart disease, etc.). Ultimately, our study included 11,126 participants (Fig 1).

### 2.2. Obtaining data on radical urbanization index and depressive symptoms

Depressive symptoms were assessed using the 10-item Center for Epidemiologic Studies Depression Scale (CES-D) [20]. The total score ranges from 0 to 30, with a score of ≥10 indicating clinically significant depressive symptoms [21]. Respondents were thus categorized into a Depressive Symptoms (DS) group (≥10) or a Non-Depressive Symptoms (NDS) group (<10), with the NDS group serving as the reference in analyses. This scale has demonstrated high validity and reliability in studies of the elderly Chinese population [22].

The radical urbanization index is calculated as the logarithm of the ratio of the urban built-up area to the total calculated urban nighttime light digital number (DN) value [23]. Built-up area data were obtained from the China City Statistical Yearbook. Nighttime light data were sourced from a harmonized DMSP-OLS-like dataset for China (1992–2019) [24], which applies different thresholds (DN ≥ 34 in eastern China and DN ≥ 25 in western China, demarcated by the “Hu Huanyong Line”) to classify urban areas. A higher index value indicates a greater degree of “land urbanization without population aggregation,” reflecting a mismatch where the expansion of built-up areas outpaces both population growth and economic activity (proxied by nighttime light).

### 2.3. Assessment of covariates

In our analysis, we included and controlled for multiple covariates, including age, gender, education level, marital status, living situation, retirement status, medical insurance coverage, alcohol consumption history, smoking history, body mass index (BMI), and the presence of hypertension, diabetes, and heart disease. Gender was categorized as male or female. Marital status was divided into “married” and “other (single, widowed/separated/divorced).” BMI was calculated using self-reported height and weight data provided by CHARLS (kg/m²). Education level was analyzed across four categories based on years of education or highest qualification: “below primary school,” “primary school,” “middle school,” and “high school or above.” Chronic diseases, primarily hypertension, diabetes, and heart disease, were determined based on respondents’ self-reports in the survey questionnaire.

### 2.4. Statistical analysis

Participant characteristics were summarized using standard deviation (SD) for continuous variables and frequency (%) for categorical variables. We employed multivariable logistic regression to estimate odds ratios (ORs) and 95% confidence intervals (CIs) for the association between the radical urbanization index (continuous) and depressive symptoms. Three models were fitted: Model 1 was unadjusted; Model 2 adjusted for age, gender, marital status, education, living situation, retirement status, medical insurance, and BMI; Model 3 further adjusted for smoking history, alcohol consumption, hypertension, diabetes, and heart disease. The reference category for the outcome was the NDS group. Subgroup analyses were conducted by stratifying the sample by gender, age (<60 vs. ≥ 60 years), BMI category (<25, 25–30, > 30 kg/m²), education level, marital status, retirement status, medical insurance, smoking history, alcohol consumption, and chronic disease status. Heterogeneity across subgroups was tested using meta-regression. The restricted cubic spline (RCS) analysis was performed with 4 knots placed at the 5th, 35th, 65th, and 95th percentiles of the radical urbanization index distribution. A two-tailed P-value <0.05 was considered statistically significant. All analyses were performed using R statistical software (version 4.2.1). No data imputation was performed for missing values.

## 3. Results

### 3.1. Baseline characteristics of the study population

In the 2011 cohort, there were a total of 11,126 participants, with 7,571 in the NDS group (Group 0) and 3,555 in the DS group (Group 1). In terms of age, the mean age in the DS group was significantly higher than that in the NDS group (60.87 years vs. 58.95 years, p < 0.001). Regarding gender distribution, the proportion of males in the DS group (37%) was lower than that in the NDS group (52%), while the proportion of females was relatively higher (63% vs. 48%, p < 0.001). In terms of educational attainment, the proportion of individuals with less than primary school education was significantly higher in the DS group (56% vs. 39%, p < 0.001), whereas a higher percentage of those with a high school education or above was observed in the NDS group (16% vs. 5.9%, p < 0.001). The proportion of retired individuals was also higher in the NDS group (18% vs. 9.8%), while the opposite was true for non-retired individuals (82% vs. 90%, p < 0.001). In terms of urban-rural differences, the proportion of urban residents in the DS group (35%) was lower than that in the NDS group (50%), while the proportion of rural residents was relatively higher (65% vs. 50%, p < 0.001).

The prevalence of hypertension was higher in the DS group (28% vs. 25%, p = 0.024), and heart disease was also more common in the DS group (17% vs. 9.8%, p < 0.001). Additionally, the analysis showed that the proportions of alcohol drinkers and smokers were both lower in the DS group compared to the NDS group. The BMI index was higher in the NDS group than in the DS group (25.59 vs. 23.07, p < 0.001), whereas the radical urbanization index was higher in the DS group than in the NDS group (−4.96 vs. −4.74, p < 0.001). (Table 1)

**Table 1:** Characteristics of study population.

Characteristic	Overall, N = 11126	NDS N = 7571	DS N = 3555	p-value
**Age**	59.52± (9.81)	58.95± (9.73)	60.87± (9.86)	<0.001
**Gender**				<0.001
Famale	5,774 (52%)	3,555 (48%)	2,219 (63%)	
Male	5,352 (48%)	4,016 (52%)	1,336 (37%)	
**Marital status**				<0.001
Others	1,381 (14%)	753 (11%)	628 (20%)	
Married	9,745 (86%)	6,818 (89%)	2,927 (80%)	
**Education**				<0.001
Below primary school	5,167 (44%)	3,101 (39%)	2,066 (56%)	
Primary school	2,412 (22%)	1,682 (22%)	730 (21%)	
Middle school	2,312 (22%)	1,747 (24%)	565 (17%)	
High school and above	1,235 (13%)	1,041 (16%)	194 (5.9%)	
**Retirement**				<0.001
No	9,814 (84%)	6,512 (82%)	3,302 (90%)	
Yes	1,312 (16%)	1,059 (18%)	253 (9.8%)	
**Medical Insurance**				0.066
No	701 (7.1%)	454 (6.6%)	247 (8.4%)	
Yes	10,425 (93%)	7,117 (93%)	3,308 (92%)	
**Residence**				<0.001
Urban	4,144 (45%)	3,100 (50%)	1,044 (35%)	
Rural	6,982 (55%)	4,471 (50%)	2,511 (65%)	
**Hypertension**				0.024
No	8,296 (74%)	5,738 (75%)	2,558 (72%)	
Yes	2,830 (26%)	1,833 (25%)	997 (28%)	
**Diabetes**				0.040
No	10,425 (93%)	7,131 (94%)	3,294 (92%)	
Yes	701 (6.7%)	440 (6.0%)	261 (8.1%)	
**Heart Disease**				<0.001
No	9,805 (88%)	6,823 (90%)	2,982 (83%)	
Yes	1,321 (12%)	748 (9.8%)	573 (17%)	
**Drinking Status**				<0.001
No	6,732 (61%)	4,405 (58%)	2,327 (67%)	
Yes	4,394 (39%)	3,166 (42%)	1,228 (33%)	
**Smoking status**				<0.001
No	6,657 (60%)	4,336 (58%)	2,321 (66%)	
Yes	4,469 (40%)	3,235 (42%)	1,234 (34%)	
**BMI**	24.84± (47.09)	25.59± (56.18)	23.07± (3.97)	<0.001
**Radical Urbanization Index**	−4.89± (0.90)	−4.96± (0.86)	−4.74± (0.97)	<0.001

### 3.2. Distribution of the radical urbanization index across Chinese Provinces and municipalities

**Fig 2:**
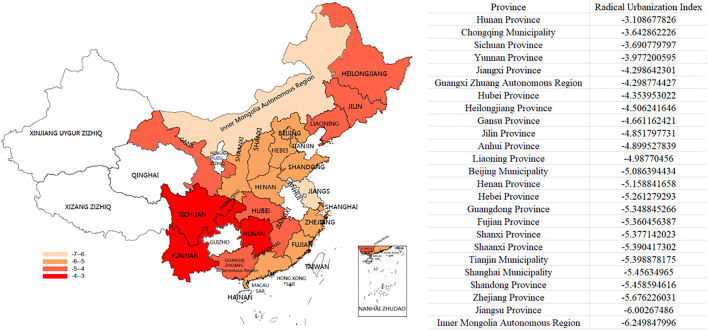
Distribution Map of the Radical Urbanization Index.

Fig 2 displayed a heatmap depicting the distribution of the radical urbanization index across various provinces in China. Heatmap was plotted by https://www.bioinformatics.com.cn (last accessed on 10 Dec 2024), an online platform for data analysis and visualization. The analysis showed that the index values are negative in the central, western, and eastern regions of China, indicating that, on average, the expansion rate of built-up areas has outpaced the growth rate of the total urban nighttime light values in these regions, with varying degrees of radical urbanization phenomena occurring in all three areas. Furthermore, when comparing the index values among these regions, the central region has the highest value, followed by the western region, and then the eastern region with the lowest. Moreover, the central and western regions have index values above the national average, while the eastern region’s index is below the national average. Therefore, the radical urbanization phenomenon is more severe in the central and western regions compared to the eastern region.

It can be observed that cities with relatively high radical urbanization indices are primarily located in the central and western regions. According to Fig 2, in 2011, Hunan, Chongqing, Sichuan, and Yunnan had the highest radical urbanization indices, with values of −3.109, −3.643, −3.691, and −3.977, respectively, while Inner Mongolia and Jiangsu had the lowest indices, at −6.003 and −6.250, respectively. Data were not recorded for nine regions, including Qinghai Province, Guizhou Province, Ningxia Hui Autonomous Region, Xinjiang Uygur Autonomous Region, Tibet Autonomous Region, Hainan Province, Macao Special Administrative Region, Hong Kong Special Administrative Region, and Taiwan Province. (Fig 2)

Heatmap was plotted by https://www.bioinformatics.com.cn/plot_basic_china_heatmap_012 (last accessed on 10 Dec 2024)

Reprinted from https://journals.plos.org/plosone/article?id=10.1371/journal.pone.0294236 under a CC BY license, with permission from Public Library of Science (PLOS), original copyright 2023.

### 3.3. Correlation between the Radical Urbanization Index and Depressive Symptoms

In Model 1, a positive correlation was observed between the radical urbanization index and depressive symptoms (OR = 1.30, 95% CI: 1.18–1.44, P < 0.001). After adjusting for age, gender, education level, marital status, living conditions, retirement status, and medical insurance coverage (Model 2), the impact of the radical urbanization index remained significant (OR = 1.24, 95% CI: 1.13–1.36, P < 0.001). Upon further adjustment for factors such as hypertension, diabetes, smoking, and alcohol consumption (Model 3), the positive correlation between the radical urbanization index and depressive symptoms remained stable (OR = 1.26, 95% CI: 1.14–1.38, P < 0.001). When categorized into quartiles based on the radical urbanization index, compared to the Q1 group, the Q2, Q3, and Q4 groups exhibited a progressively increasing risk of depressive symptoms in both Model 2 and Model 3 (P for trend < 0.001). Moreover, the differences between the Q3 and Q4 groups were statistically significant. (Table 2)

**Table 2:** Association between Radical Urbanization Index. group and depression (Logistic regression).

	Model 1			Model 2			Model 3		
**OR** ^1^	**95% CI** ^1^	**p-value**	**OR** ^1^	**95% CI** ^1^	**p-value**	**OR** ^1^	**95% CI** ^1^	**p-value**
**Radical Urbanization Index**	1.30	1.18, 1.44	<0.001	1.24	1.13, 1.36	<0.001	1.26	1.14, 1.38	<0.001
Q1	—	—		—	—		—	—	
Q2	1.13	0.82, 1.56	0.4	1.26	0.93, 1.70	0.13	1.23	0.92, 1.65	0.2
Q3	1.28	0.95, 1.74	0.11	1.43	1.10, 1.86	0.008	1.42	1.09, 1.84	0.009
Q4	1.80	1.34, 2.41	<0.001	1.65	1.25, 2.18	<0.001	1.68	1.27, 2.21	<0.001
P for trend		<0.001			<0.001			<0.001	

Model 1: Unadjusted

Model 2: Adjusted for age, gender, education level, marital status, living conditions, retirement status, and medical insurance coverage

Model 3: Adjusted for age, gender, education level, marital status, living conditions, retirement status, medical insurance coverage, hypertension, diabetes, smoking, alcohol consumption, etc.

Model fit was assessed using the Akaike Information Criterion (AIC) and Bayesian Information Criterion (BIC): Model 1 (AIC: [13454.27], BIC: [13492.01]), Model 2 (AIC: [12749.12], BIC: [12960.78]), Model 3 (AIC: [12595.55], BIC: [12892.08]).

### 3.4. Nonlinear Relationship between the Radical Urbanization Index and Depression

**Fig 3:**
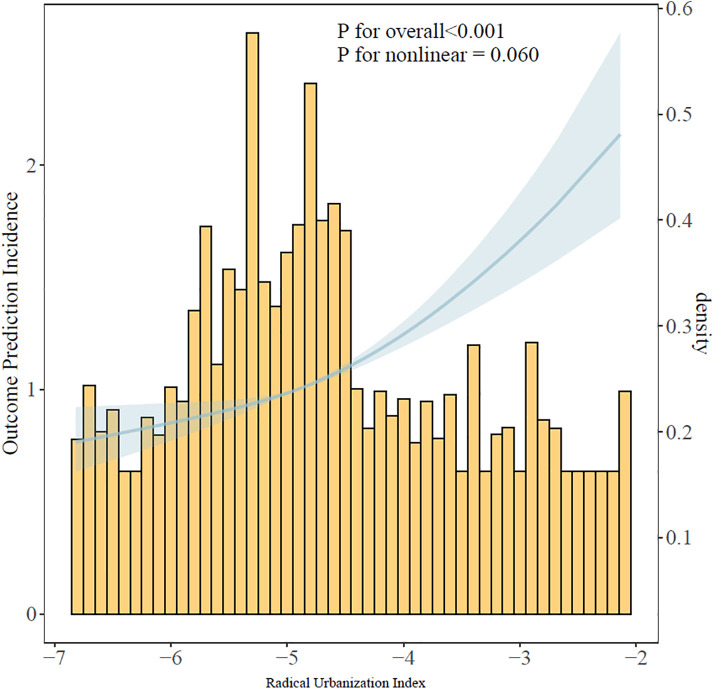
Presents an analysis of the association between the radical urbanization index and depressive symptoms using restricted cubic spline (RCS) functions. The blue line represents the odds ratio, and the blue shaded area indicates the 95% confidence interval. No significant nonlinear trend was observed (P for nonlinearity = 0.060). The logistic regression was adjusted for age, gender, education level, marital status, living conditions, retirement status, medical insurance coverage, alcohol consumption history, smoking history, body mass index (BMI), and the presence of hypertension, diabetes, and heart disease.

Through restricted cubic spline (RCS) analysis, the analysis showed a significant positive correlation between the radical urbanization index and the risk of depression (overall P < 0.001). However, no significant nonlinear trend was observed between the radical urbanization index and depressive symptoms (P for nonlinearity = 0.060) (Fig 3). The RCS analysis revealed that for every 1-unit increase in the urbanization index, the risk of depression increased by 26% (95% CI: 1.14–1.38). Although the nonlinearity test did not reach statistical significance, the curve shape suggested an accelerated upward trend in risk at high levels of radical urbanization (index > −4).

### 3.5. Subgroup analysis of the association between the radical urbanization index and depressive symptoms

**Fig 4:**
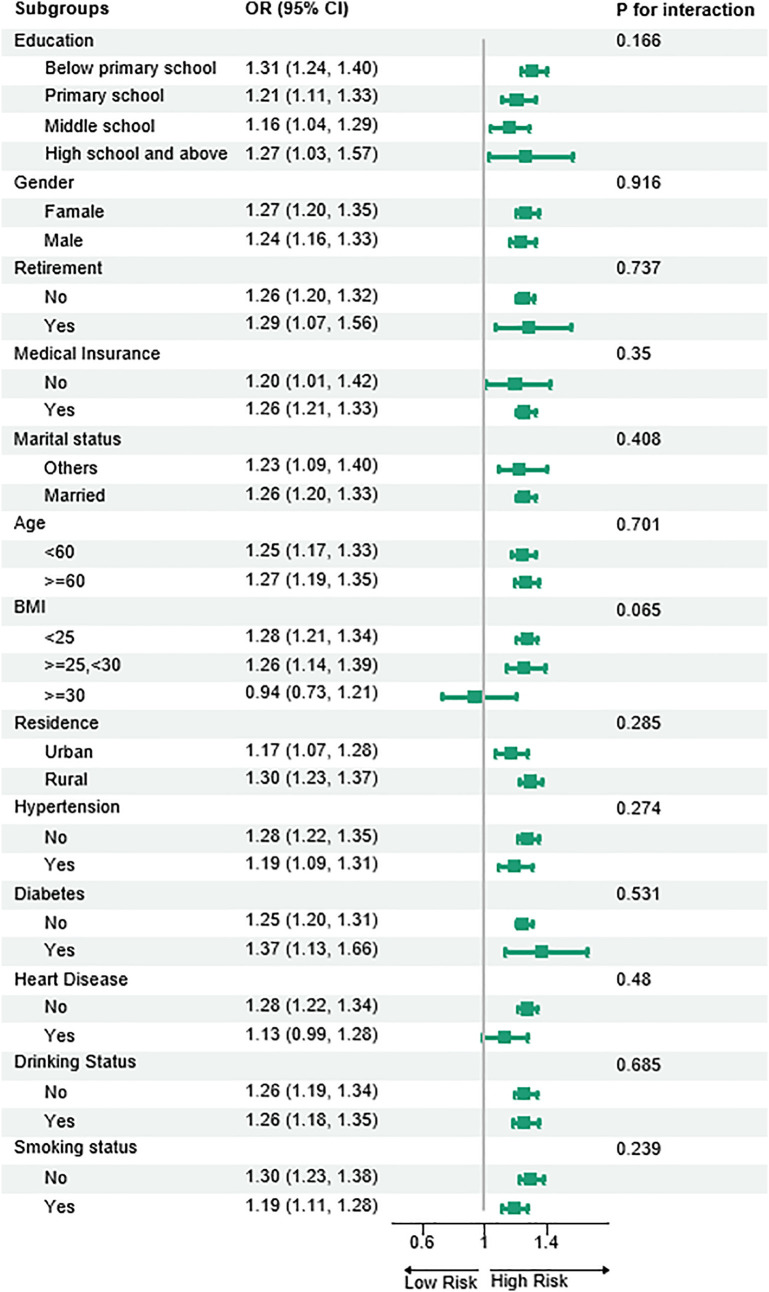
Subgroup analyses investigating the interaction between patient characteristics and the association between Radical Urbanization Index and depression.

The positive correlation between the radical urbanization index and depressive symptoms was generally observed across all subgroups (OR > 1), but with some heterogeneity. A marginal interaction was noted in the BMI subgroup (P = 0.085), where the association was not significant among obese individuals (BMI ≥ 30; OR = 0.94, 95% CI: 0.73–1.27), while the risk increased significantly among non-obese individuals (BMI < 25: OR = 1.28; BMI 25–30: OR = 1.26). Higher point estimates were observed in subgroups with lower education levels (below primary school: OR = 1.31), rural residents (OR = 1.30), and non-smokers (OR = 1.30). No significant interactions were found for smoking status (P = 0.239), place of residence (P = 0.285), education level (P = 0.166), gender (P = 0.916), age (P = 0.701), or most chronic disease subgroups (P > 0.05). Notably, the association did not reach statistical significance among patients with heart disease (OR = 1.13, 95% CI: 0.99–1.29), while a significant positive correlation persisted in all other subgroups. (Fig 4)

### 3.6. Performance Comparison of Depressive Symptom Classification Model Construction

**Fig 5:**
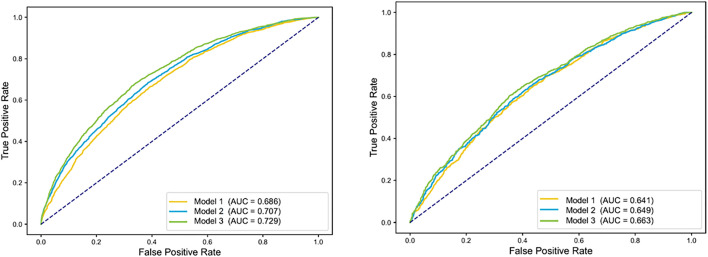
Comparative ROC curves of three classification models. The x-axis represents the False Positive Rate (FPR), with a scale ranging from 0.0 to 1.0, and the y-axis represents the True Positive Rate (TPR). **A)** Performance metrics of the three Train Set classification models: Model 1 (AUC = 0.626), Model 2 (AUC = 0.707), and Model 3 (AUC = 0.729); **B)** Performance metrics of the three Test Set classification models: Model 1 (AUC = 0.641), Model 2 (AUC = 0.649), and Model 3 (AUC = 0.683).

In the training set, Model 3 (the model incorporating the Radical Urbanization Index) demonstrated higher accuracy compared to Model 1 (the demographic-based model) and Model 2 (the model excluding the Radical Urbanization Index) (0.712 vs. 0.703 vs. 0.691). Similarly, in the test set, Model 3 also outperformed Model 1 and Model 2 (0.684 vs. 0.682 vs. 0.675). Furthermore, in the test set, the sensitivity of the model incorporating the Radical Urbanization Index was higher than that of the other two models. Model 3 exhibited the best performance in both the training and test sets (test AUC = 0.663), but it suffered from relatively severe overfitting (training AUC = 0.771 → test AUC = 0.663). (Table 3) (Fig 5).

**Table 3:** Characteristics of study population.

	Model	Model1	Model2	Model3
**Train Set**	**Accuracy**	0.691	0.708	0.728
	**Sensitivity**	0.693	0.711	0.727
	**Specificity**	0.633	0.673	0.737
	**AUC** **(95% CI)**	0.685 (0.683,0.687)	0.719 (0.716,0.722)	0.771 (0.768,0.774)
**Test Set**	**Accuracy**	0.674	0.68	0.691
	**Sensitivity**	0.675	0.686	0.698
	**Specificity**	0.5	0.562	0.605
	**AUC** **(95% CI)**	0.639 (0.636,0.642)	0.649 (0.645,0.653)	0.663 (0.659,0.667)

Model 1: Model with demographic.

Model 2: Model without Radical Urbanization Index.

Model 3: Model with Radical Urbanization Index.

## 4. Discussion

This study is the first to employ the “Radical Urbanization Index” to investigate the association between the spatial mismatch in urban development and the risk of depression among middle-aged and elderly individuals in China. We found that a higher Radical Urbanization Index was independently associated with an increased risk of depressive symptoms. Specifically, for every 1-unit increase in the index, the odds of depression rose by 26% (OR = 1.26, 95% CI: 1.14–1.38). Although modest, this association poses a non-trivial public health concern given the massive scale of populations exposed to radical urbanization in China. This association was particularly pronounced in central and western regions and exhibited a dose-response relationship; individuals in the highest quartile (Q4) of the index had 1.68 times higher odds of depression compared to those in the lowest quartile (Q1). The association was also stronger among subgroups with lower education levels (OR = 1.31), rural residents (OR = 1.30), and non-obese individuals, whereas no significant association was observed among obese individuals (BMI ≥ 30) or those with heart disease. Although this large-sample cross-sectional analysis did not detect a significant nonlinear trend, the fitted curve suggested a potential acceleration in risk at very high levels of radical urbanization (index > −4).

The observed association can be interpreted through the lens of China’s distinct urbanization pattern. Urbanization is a multi-dimensional process involving land, population, and industry. However, China’s rapid development has often prioritized physical land expansion over the concurrent growth of population and industries—a pattern termed the radical model of urbanization.Radical urbanization arises from human factors, such as improper urban planning and construction leading to severe disintegration among populations, production, and towns, resulting in towns or areas with sparse populations, lack of industries, and economic stagnation [25], More conspicuous features include high vacancy rates of buildings and low utilization rates of infrastructure. The expansion rate of built-up areas outpaces the growth rate of the urban population, manifesting as insufficient nighttime lighting and economic activity [26]，pointing to inefficient and disorderly urban expansion in Chinese cities. We propose that the tangible manifestations of radical urbanization, such as building vacancies and underutilized infrastructure, may contribute to depression risk by eroding community cohesion, limiting healthcare access, and fostering economic uncertainty. This provides crucial empirical evidence from the context of rapid urbanization in China for the “built environment-mental health” theoretical framework [27,28]. Furthermore, by incorporating the Radical Urbanization Index into depression prediction models (with the AUC increasing from 0.649 to 0.663), we demonstrate the incremental predictive value of the index, surpassing the limitations of traditional regression analysis. This study offers quantifiable mental health early warning indicators for urban planning departments, promoting a shift in public health interventions from “individual treatment” to “environmental regulation.”

Our findings contribute to clarifying the complex and debated relationship between urbanization and mental health. There has been ongoing debate about whether urbanization leads to depression, particularly among the elderly amid the rising global trend of aging. Existing studies on urbanization and depression present two opposing viewpoints: Some research suggests that cities reduce the risk of depression through the aggregation of high-quality medical resources, diversity of social networks, and richness of cultural stimuli. Using the original CHARLS dataset from China, some studies have analyzed differences in the incidence of depression between urban and rural areas, observing a protective effect of urbanization against depression [6,29]. A study published in PNAS in the United States revealed that large cities, by providing more opportunities for social connections, reduce the risk of depression among socially isolated individuals, demonstrating a “sublinear relationship between the number of depression cases and city size” [30]. Conversely, other studies emphasize that the urban environment increases the risk of depression through social isolation, exposure to environmental pollutants (such as air pollution and noise), and life stress events (such as highly competitive environments) [31–33]. The recently proposed Urban Resilience Index demonstrates that high levels of urban resilience can significantly reduce the risk of depression symptoms among middle-aged and elderly individuals through a multidimensional analysis [34]. Additionally, some recent studies suggest that the ultimate nature of depression among rural and urban residents is homogeneous, arguing that urbanization may not affect depression. They propose that cities have both protective and harmful effects on depression, with the protective effects only emerging when central cities, suburbs, and rural areas are combined [35,36]. By moving beyond the simple urban-rural dichotomy and introducing the Radical Urbanization Index, our study shifts the focus from the extent to the quality of urbanization. The inefficient sprawling urban form revealed by this index constitutes significant environmental pressure [37], severely mismatching the needs of middle-aged and elderly residents for accessible services, social engagement, and economic stability. The physical decay and lack of vitality in highly radicalized areas may well undermine the foundations of community life. Building vacancies and idle public spaces are not merely physical phenomena; they symbolize economic stagnation and diminish opportunities for spontaneous social interactions that build trust and mutual aid. Weakened community cohesion and collective efficacy exacerbate feelings of social isolation and helplessness—powerful precursors to depression [38]. Subgroup findings further validate this perspective: rural residents and those with lower educational attainment are disproportionately affected. These groups, lacking personal resources, may be more vulnerable to such environmental pressures. Thus, the radical urbanization index quantifies the rupture in the positive feedback loop that should exist between urban density and social interaction.

Several limitations of this study should be considered. Firstly, the cross-sectional research design precludes causal inference. Despite adjustments for numerous covariates, residual confounding caused by unmeasured factors may still exist. Secondly, this study focuses on the middle-aged and elderly population in China, which may limit the generalizability of the findings to other age groups or cultural backgrounds. Thirdly, the city-level Radical Urbanization Index may fail to capture finer-scale variations within cities; future research could employ multi-scale indicators, such as lighting density at the community level or street-view imagery. Fourthly, the specific mechanisms underlying the association between this index and depression remain unclear. Future studies should directly explore potential pathways, including environmental stressors and neurobiological responses. Finally, the city-level Radical Urbanization Index may not adequately capture intra-city variations, and the ecological research design does not allow for individual-level inferences. Future research could utilize multi-level modeling approaches or community-level indicators to mitigate ecological fallacy.

## 5. Conclusion

This study innovatively links the “Radical Urbanization Index”—a measure of the spatial mismatch between land expansion and economic activity—with depression risk among middle-aged and elderly Chinese, identifying it as a significant environmental correlate. Our findings bridge a critical theoretical gap by demonstrating that the model of “land urbanization” outpacing “population urbanization” (the radical model) may impose tangible costs on residents’ mental health. To advance this field, subsequent research should prioritize establishing causality through prospective designs and unraveling the underlying mechanisms. Ultimately, these efforts can inform integrated public health strategies that coordinate spatial planning, community governance, and individual services, steering urbanization toward a more sustainable and healthy model of human-land coordination—a balanced development of population, economy, and land use.
