# Correction: The impact of the radical urbanization index on depression risk among middle-aged and elderly Chinese: Evidence from a large-scale cross-sectional study

**DOI:** 10.1371/journal.pone.0348900

**Published:** 2026-05-05

**Authors:** Yuting Li, Diege Long, Zhen Ye

There is an error in affiliation 1 for author Yuting Li. The correct affiliation 1 is: The Traditional Chinese Medicine Hospital of Longquanyi, Chengdu.
